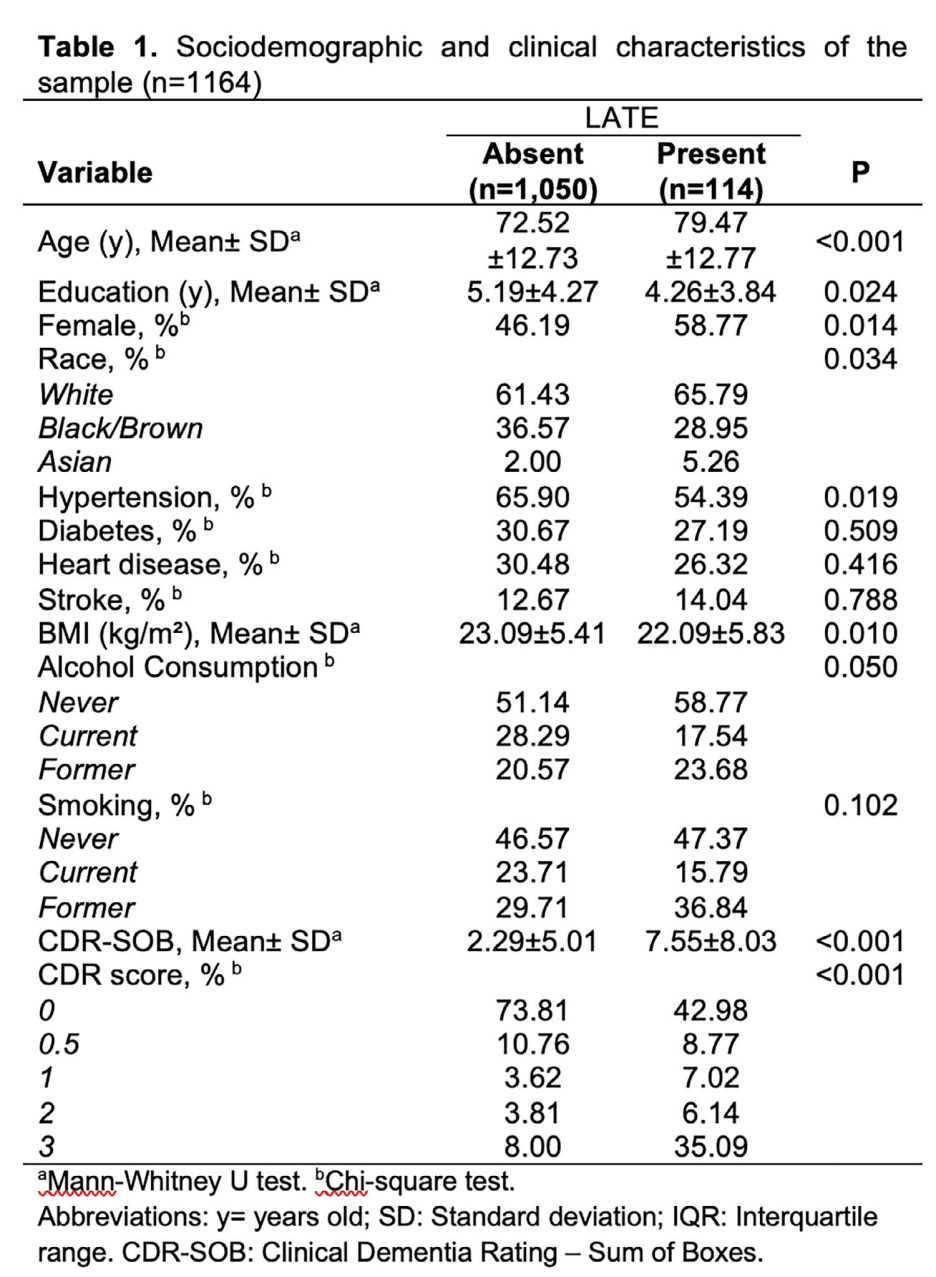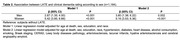# The sex related association between TDP‐43 and cognitive function: a population‐based autopsy study

**DOI:** 10.1002/alz.090471

**Published:** 2025-01-03

**Authors:** Caroline Matos Silva, Alberto Fernando Oliveira Justo, Renata Elaine Paraizo Leite, Vitor Ribeiro Paes, Lea T. Grinberg, Carlos Augusto Pasquallucci, Wilson Jacob‐Filho, Ricardo Nitrini, Claudia Kimie Suemoto

**Affiliations:** ^1^ Biobank for Aging Studies of the University of São Paulo, São Paulo Brazil; ^2^ University of São Paulo Medical School, São Paulo, São Paulo Brazil; ^3^ Memory and Aging Center, University of California, San Francisco, CA USA

## Abstract

**Background:**

Transactive DNA‐binding protein 43 (TDP‐43) proteinopathy is associated with neurodegeneration, including LATE and linked to cognitive deterioration. While some research suggests a higher prevalence of TDP‐43 in women, no differences have been identified among racial groups. Nonetheless, the influence of gender on cognition within the context of TDP‐43 remains uncertain. Our objective was to investigate gender and racial distinctions in the relationship between TDP‐43 presence and cognitive function.

**Method:**

Cross‐sectional study in a population‐based sample of 1,164 Brazilians from the Biobank for Aging Studies of the University of Sao Paulo Medical School. We used linear regression to evaluate the association of progressive LATE stages with cognitive abilities evaluated by Clinical Dementia Rating Sum of Boxes (CDR‐SOB). Models were adjusted for sociodemographic (age at death, sex, education, and race), clinical variables (hypertension, diabetes, heart disease, stroke, alcohol consumption, smoking, and body mass index) and neuropathological lesions (Braak staging, CERAD, Lewy body diseases pathology, lacunar infarcts, hyaline arteriolosclerosis and cerebral angiopathy amyloid). Additionally, we investigated the interaction of TDP‐43 pathology with race (white and black) and sex on CDR‐SOB.

**Result:**

Sociodemographic and clinical variables were different between the participants with and without LATE (Table 1). Adjusted analysis, accounting for sociodemographic and clinical factors, revealed a significant association between the presence of LATE and impaired cognitive abilities (β = 5.13, 95% CI = 3.63;6.33, p<0.001). We did not find an interaction between race and LATE (p = 0.73). However, we found a significant interaction between TDP‐43 and sex (p = 0.001) (Figure 1). Upon conducting a stratified analysis, LATE had a more pronounced impact on cognitive abilities in women than in men (Men: β = 2.56, 95% CI = 1.26;3.86, p<0.001; Women: β = 4.94, 95% CI = 3.52;6.36, p for interaction <0.001).

**Conclusion:**

The presence of LATE is associated with poor cognition, particularly in women.